# Supplementary material for: Improved regression in ratio type estimators based on robust M-estimation
Source: PLoS One. 2022 Dec 12;17(12):e0278868. doi: 10.1371/journal.pone.0278868 (PMC9744333; doi:10.1371/journal.pone.0278868)

PLOS ONE

Improved Regression in Ratio Type Estimators Based on Robust M-Estimation

--Manuscript Draft--

**Manuscript Number:**	PONE-D-22-27634
**Article Type:**	Research Article
**Full Title:**	Improved Regression in Ratio Type Estimators Based on Robust M-Estimation
**Short Title:**	Improved Regression in Ratio Type Estimators
**Corresponding Author:**	KHALID UL ISLAM RATHER, ph.D.<br>Sher-e-Kashmir University of Agricultural Sciences and Technology of Kashmir<br>pulwama, JAMMU & KASHMIR INDIA
**Keywords:**	ratio estimator; robust estimation; m-estimator; simple random sampling; efficiency
**Abstract:**	In this article, a new robust ratio type estimator using the Uk's redescending M-estimator is proposed for the estimation of the finite population mean in the simple random sampling (SRS) when there are outliers in the dataset. The mean square error (MSE) equation of the proposed estimator is obtained using the first order of approximation and it has been compared with the traditional ratio-type estimators in the literature, robust regression estimators, and other existing redescending M-estimators. A real-life data and simulation study are used to justify the efficiency of the proposed estimators. It has been shown that the proposed estimator is more efficient than other estimators in the literature on both simulation and real data studies.
**Order of Authors:**	KHALID UL ISLAM RATHER, ph.D.
	Eda Gizem Koçyiğit, PhD
	Ronald Onyango, PhD
	Cem Kadılar
**Opposed Reviewers:**	
**Additional Information:**	
**Question**	**Response**
**Financial Disclosure**<br><br>Enter a financial disclosure statement that describes the sources of funding for the work included in this submission. Review the submission guidelines for detailed requirements. View published research articles from *PLOS ONE* for specific examples.<br><br>This statement is required for submission and **will appear in the published article** if the submission is accepted. Please make sure it is accurate.	NO

Powered by Editorial Manager® and ProduXion Manager® from Aries Systems Corporation

**Unfunded studies**<br>Enter: *The author(s) received no specific funding for this work.*<br><br>**Funded studies**<br>Enter a statement with the following details:<br>• Initials of the authors who received each award<br>• Grant numbers awarded to each author<br>• The full name of each funder<br>• URL of each funder website<br>• Did the sponsors or funders play any role in the study design, data collection and analysis, decision to publish, or preparation of the manuscript?<br>• **NO** - Include this sentence at the end of your statement: *The funders had no role in study design, data collection and analysis, decision to publish, or preparation of the manuscript.*<br>• **YES** - Specify the role(s) played.<br><br>* typeset	
**Competing Interests**<br><br>Use the instructions below to enter a competing interest statement for this submission. On behalf of all authors, disclose any competing interests that could be perceived to bias this work—acknowledging all financial support and any other relevant financial or non-financial competing interests.<br><br>This statement is required for submission and **will appear in the published article** if the submission is accepted. Please make sure it is accurate and that any funding sources listed in your Funding Information later in the submission form are also declared in your Financial Disclosure statement.<br><br>View published research articles from *PLOS ONE* for specific examples.	The authors have declared that no competing interests exist

**NO authors have competing interests**<br><br>Enter: *The authors have declared that no competing interests exist.*<br><br>**Authors with competing interests**<br><br>Enter competing interest details beginning with this statement:<br><br>*I have read the journal's policy and the authors of this manuscript have the following competing interests: [insert competing interests here]*<br><br>* typeset	
**Ethics Statement**<br><br>Enter an ethics statement for this submission. This statement is required if the study involved:<br><br>• Human participants<br>• Human specimens or tissue<br>• Vertebrate animals or cephalopods<br>• Vertebrate embryos or tissues<br>• Field research<br><br>Write "N/A" if the submission does not require an ethics statement.<br><br>General guidance is provided below. Consult the submission guidelines for detailed instructions. **Make sure that all information entered here is included in the Methods section of the manuscript.**	N/A

**Format for specific study types**<br><br>**Human Subject Research (involving human participants and/or tissue)**<br>• Give the name of the institutional review board or ethics committee that approved the study<br>• Include the approval number and/or a statement indicating approval of this research<br>• Indicate the form of consent obtained (written/oral) or the reason that consent was not obtained (e.g. the data were analyzed anonymously)<br><br>**Animal Research (involving vertebrate animals, embryos or tissues)**<br>• Provide the name of the Institutional Animal Care and Use Committee (IACUC) or other relevant ethics board that reviewed the study protocol, and indicate whether they approved this research or granted a formal waiver of ethical approval<br>• Include an approval number if one was obtained<br>• If the study involved *non-human primates*, add *additional details* about animal welfare and steps taken to ameliorate suffering<br>• If anesthesia, euthanasia, or any kind of animal sacrifice is part of the study, include briefly which substances and/or methods were applied<br><br>**Field Research**<br><br>Include the following details if this study involves the collection of plant, animal, or other materials from a natural setting:<br>• Field permit number<br>• Name of the institution or relevant body that granted permission	
**Data Availability**<br><br>Authors are required to make all data underlying the findings described fully available, without restriction, and from the time of publication. PLOS allows rare exceptions to address legal and ethical concerns. See the PLOS Data Policy and FAQ for detailed information.	Yes - all data are fully available without restriction

A Data Availability Statement describing where the data can be found is required at submission. Your answers to this question constitute the Data Availability Statement and **will be published in the article**, if accepted.<br><br>**Important:** Stating 'data available on request from the author' is not sufficient. If your data are only available upon request, select 'No' for the first question and explain your exceptional situation in the text box.<br><br>Do the authors confirm that all data underlying the findings described in their manuscript are fully available without restriction?	
**Describe where the data may be found in full sentences. If you are copying our sample text, replace any instances of XXX with the appropriate details.**<br><br>• If the data are **held or will be held in a public repository**, include URLs, accession numbers or DOIs. If this information will only be available after acceptance, indicate this by ticking the box below. For example: *All XXX files are available from the XXX database (accession number(s) XXX, XXX.*).<br>• If the data are all contained **within the manuscript and/or Supporting Information files**, enter the following: *All relevant data are within the manuscript and its Supporting Information files.*<br>• If neither of these applies but you are able to provide **details of access elsewhere**, with or without limitations, please do so. For example:<br><br>*Data cannot be shared publicly because of [XXX]. Data are available from the XXX Institutional Data Access / Ethics Committee (contact via XXX) for researchers who meet the criteria for access to confidential data.*<br><br>*The data underlying the results presented in the study are available from (include the name of the third party*	All relevant data are within the manuscript.

and contact information or URL).<br>• This text is appropriate if the data are owned by a third party and authors do not have permission to share the data.<br><br>* typeset	
Additional data availability information:	

Powered by Editorial Manager® and ProduXion Manager® from Aries Systems Corporation

COVER LETTER FOR SUBMISSION OF NEW MANUSCRIPT

Subject: **SUBMISSION OF NEW MANUSCRIPT FOR EVALUATION**

I am enclosing herewith a manuscript entitled “Improved regression in ratio type estimators based on robust regression m-estimation” submitted for possible evaluation.

With the submission of this manuscript, I would like to undertake that the above mentioned manuscript has not been published elsewhere, accepted for publication elsewhere or under editorial review for publication elsewhere. This research did not receive any specific grant from funding agencies in the public, commercial, or not-for-profit sectors.

Yours faithfully,

Ronald Onyango
assangaronald@gmail.com

Manuscript with author details Click here to access/download;Manuscript;manuscript.docx

Improved Regression in Ratio Type Estimators Based on Robust M-Estimation

Khalid Ul Islam Rather[1*], Eda Gizem Koçyiğit[2¶], Ronald Onyango[3&] and Cem Kadilar[4&]

[1*] *Division of Statistics and Computer Science, Main Campus SKUAST-J,Chatha Jammu-180009, India. ORCHID ID: 0000-0002-4631-5652*

[2] *Department of Statistics, Dokuz Eylül University, Buca 35390 İzmir, Türkiye. ORCHID ID: 0000-0002-0774-1376*

[3] *Department of Applied Statistics, Financial Mathematics, and Actuarial Science, Jaramogi Oginga Odinga University of Science and Technology, Kenya. ORCHID ID:0000-0002-8652-3819*

[4] *Department of Statistics, Hacettepe University, Beytepe, Ankara 06800, Türkiye. ORCHID ID: 0000-0003-4950-9660*

[*] Corresponding author

E-mail address: khalidstat34@gmail.com

[¶] These authors contributed equally to this work.

[&] These authors also contributed to this work.

Abstract

In this article, a new robust ratio type estimator using the Uk's redescending M-estimator is proposed for the estimation of the finite population mean in the simple random sampling (SRS) when there are outliers in the dataset. The mean square error (MSE) equation of the proposed estimator is obtained using the first order of approximation and it has been compared with the traditional ratio-type estimators in the literature, robust regression estimators, and other existing redescending M-estimators. A real-life data and simulation study are used to justify the efficiency of the proposed estimators. It has been shown that the proposed estimator is more efficient than other estimators in the literature on both simulation and real data studies.

1. Introduction

Outliers are observations that behave differently from the majority in datasets and often can significantly affect statistics. In sampling studies, however, the presence of outliers cannot be easily detected, since the entire population cannot always be reached. Especially in methods that need to work with small sample sizes, the efficiency of the estimation decreases if an outlier observation is taken into the sample.

To reduce the consequences of an outlier(s) in the real data, robust regression methods are generally used. M-estimators are used as a robust replacement for the general classical estimators utilized in the field of statistics. To overcome the problem of outliers efficiently as compared to other robust estimation methods, the Uk's redescending M-estimator is proposed [1]. The outliers in the data mainly affect the traditional estimation methods and reduce their efficiencies. In fact, the performance of the ordinary least square (OLS) estimators reduces in the presence of outliers. Therefore, numerous redescending M-estimation methods have been developed to

control the consequences of outliers and to improve the efficiency of estimation procedure, including [2-12]. This study aims to reduce the effect of outliers by developing a new ratio-type redescending M-estimator based on the Uk's redescending M-estimator (URME) that may improve the efficiency of URME and provide a perfect estimation.

This article is organized as follows: Section 2 introduces the traditional ratio estimators based on previous estimators and some existing M-estimators in the literature. In Section 3, we give brief information about Uk's redescending M-Estimator and then present the proposed estimator. In addition, efficiency comparisons of the proposed estimator are given in the last part of this section. Section 4 calculates the relative efficiencies of the estimators and compares these estimators with each other in theory and in the application by simulation and real data, respectively. Lastly, Section 5 concludes and offers for the future studies.

2. Existing estimators in the literature

2.1. Kadilar and Cingi (2004) estimators

In the simple random sampling, Kadilar and Cingi [13] introduced ratio estimators by adapting the traditional estimators and other ratio-type estimators in literature [13-14]. On the base of MSE equations and numerical illustrations, it was proved that the efficiencies of the proposed estimators are better than OLS estimators. These estimators are

$$\bar{y}_{KC_j} = \frac{\bar{y}+b\left(\bar{X}-\bar{x}\right)}{\left(\beta_j \bar{x}+\gamma_i\right)}\left(\beta_j \bar{X}+\gamma_i\right),\ j=1,2,3,4,5 \qquad (1)$$

where $\bar{y}$ is the observed sample mean of the study variable and $\bar{x}$ is the sample mean of the auxiliary variable. Also, $\beta_1 = 1$ and $\gamma_1 = 0$, $\beta_2 = 1$ and $\gamma_2 = C_x$, $\beta_3 = 1$ and $\gamma_3 = \beta_2(x), \beta_4 = \beta_2(x)$ and $\gamma_4 = C_x$, $\beta_5 = C_x$ and $\gamma_5 = \beta_2(x)$. Here, $\beta_2(x)$ and C_x are the coefficient of kurtosis and coefficient of variation of the auxiliary variable, respectively. Note that b is the slope coefficient derived from the OLS estimation. However, the occurrence of outliers vanishes the productivity and proficiency of these estimators. Therefore, Kadilar *et al*. [15] proposed new ratio estimators for the efficient estimation of the population mean. The MSE equation of $\bar{y}_{KC_j}$ is as follows:

$$MSE\left(\bar{y}_{KC_j}\right) \cong \theta\left(R_j^2 S_x^2 + 2BR_j S_x^2 + B^2 S_x^2 - 2R_j S_{xy} - 2BS_{xy} + S_y^2\right) \text{ for } j=1,2,3,4,5 \tag{2}$$

where $\theta = \frac{1-f}{n}$, $f = \frac{n}{N}$ and B is obtained by an expected value of b such that E(b)=B.

$$R_j = \frac{\beta_j \bar{Y}}{\beta_j \bar{X} + \gamma_j} \text{ for } j=1,2,3,4,5 \tag{3}$$

It is worth to note that the ratio estimator, given in Eq (1), has higher potentiality and proficiency in the existence of outliers than that of other traditional estimators in the literature [16-17].

2.2. Kadilar *et al*. (2007) Huber M-estimators

For the regression analysis, numerous methods have been introduced in the literature to deal with the problem of outliers in the data. Such estimators were initially developed by Huber [9], but later on, Kadilar *et al*. [15] gave emphasis to these estimators by using the robust regression as a substitute of OLS. The estimators were named as Huber M-estimators (HM) and they were given as

$$\bar{y}_{HM_j} = \frac{\bar{y} + b_{HM}\left(\bar{X} - \bar{x}\right)}{\left(\beta_j \bar{x} + \gamma_i\right)}\left(\beta_j \bar{X} + \gamma_i\right), \; j = 1, 2, 3, 4, 5 \tag{4}$$

where b_{HM} is the slope coefficient of the robust regression M-estimators given by Huber [9]. The design of Huber's function $\rho\left(r_j\right)$ is given by

$$\rho\left(r_j\right) = \begin{cases} \frac{r^2}{2} & for \; |r| \le c \\ c|r| - \frac{c^2}{2} & for \; |r| > c \end{cases} \tag{5}$$

where r is the random error following the OLS method while c is the tuning constant.

The advised value of c from the Huber [9] is one and half times of the estimated standard deviation of error. The MSE equation of the M-estimators is given as follows:

$$MSE\left(\bar{y}_{HM_j}\right) \cong \theta\left(R_j^2 S_x^2 + 2B_{HM} R_j S_x^2 + B_{HM}^2 S_x^2 - 2R_j S_{xy} - 2B_{HM} S_{xy} + S_y^2\right) \quad \text{for } j = 1, 2, 3, 4, 5 \tag{6}$$

where B_{HM} is the expected slope coefficient of b. The MSE for the estimators, given in Eq (6), can also be obtained by replacing B in Eq (2) by B_{HM}. The MSE computed for M-estimators are relatively more efficient as compared to the OLS estimators.

2.3. Raza *et al.* (2019) estimators

Raza *et al.* [18] proposed ratio estimators based on the newly developed robust redescending M-estimator. The redescending M-estimators (RM) are given by

$$\bar{y}_{RM_j} = \frac{\bar{y} + b_{RM}\left(\bar{X} - \bar{x}\right)}{\left(\beta_j \bar{x} + \gamma_i\right)}\left(\beta_j \bar{X} + \gamma_i\right), \; j = 1, 2, 3, 4, 5 \tag{7}$$

where b_{RM} is the slope coefficient of the redescending M-estimators given by Raza *et al.* [18]. The design of the Raza's objective function $\rho\left(r_j\right)$ is described as

$$\rho_1\left(r_j\right)=\frac{v^2}{2c}\left\{1-\left[1+\left(\frac{r}{v}\right)^2\right]^{-c}\right\} \; for \; |r|\geq 0 \tag{8}$$

where c and v are tuning constants. For the current study, optimum values of the tuning constant are c=2.5 and v=8. The b_{RM} redescending M-estimator is used in the MSE equation of the ratio estimators in Eq (7) as follows:

$$MSE\left(\bar{y}_{RM_j}\right)\cong\theta\left(R_j^2S_x^2+2B_{RM}R_jS_x^2+B_{RM}^2S_x^2-2R_jS_{xy}-2B_{RM}S_{xy}+S_y^2\right) \\ \text{for } j=1,2,3,4,5 \tag{9}$$

2.4. Noor-ul-Amin *et al.* (2020) estimators

Noor-ul-Amin *et al.* [19] proposed another ratio estimator using the robust M-estimators and named it as redescending M-estimators under the different objective function given by

$$\bar{y}_{NM_j}=\frac{\bar{y}+b_{NM}\left(\bar{X}-\bar{x}\right)}{\left(\beta_j\bar{x}+\gamma_i\right)}\left(\beta_j\bar{X}+\gamma_i\right),\; j=1,2,3,4,5 \tag{10}$$

where b_{NM} is the slope coefficient of the redescending M-estimators given by Noor-ul-Amin *et al.* [19]. The design of the Noor objective function $\rho_2\left(r_j\right)$ is described as

$$\rho_2\left(r_j\right)=\frac{c^2}{4}\left[\frac{\tan^{-1}\left(\frac{2r}{c}\right)^2}{4}+\frac{r^2c^2}{c^4+16r^4}\right] \; for \; |r|\geq 0 \tag{11}$$

The MSE equation of the ratio estimators, given in Eq (10), is calculated with the same

method as that given before.

$$MSE\left(\bar{y}_{NM_j}\right) \cong \theta\left(R_j^2 S_x^2 + 2B_{NM} R_j S_x^2 + B_{NM}^2 S_x^2 - 2R_j S_{xy} - 2B_{NM} S_{xy} + S_y^2\right) \quad \text{for } j = 1,2,3,4,5 \tag{12}$$

3. Proposed ratio estimators based on Uk's redescending M-estimator

3.1. Uk's redescending M-estimator

The proposed estimator is also known as Uk's redescending M-estimator. The M-estimator of β is defined by the following objective function:

$$Minimize_{\hat{\beta}} \sum_{i=1}^{n} \rho\left(r_i\right) \tag{13}$$

where $r_i = y_i - \beta x_i$ represents the residuals. An objective function must fulfil the following standard properties:

- $\rho(0) = 0$
- $\rho(r_i) \geq 0$
- $\rho(r_i) = \rho(-r_i)$
- $\rho(r_i) \geq \rho(r_j)$ for $|r_i| \geq |r_j|$
- ρ is differentiable

M-estimator is called a redescending M-estimator if it fulfils the standard related properties and the derivative of its objective function is ψ-function. Differentiating Eq (13) with respect to $\hat{\beta}_j$ we obtain $\psi\left(r_i\right)$ function as follows:

$$\sum_{i=1}^{n} \psi(r_i) X_i = 0 \qquad (14)$$

Dividing $\psi(r_i)$ by r_i we obtain the weight function as

$$\sum_{i=1}^{n} w(r_i) X_i = \frac{\sum_{i=1}^{n} \psi(r_i) X_i}{r} \qquad (15)$$

On the base of procedure, defined in Eqs (5), (8) and (11), a redescending M-estimator is proposed with the aid of [1]. The objective function of the proposed estimator is

$$\rho(r) = \begin{cases} \frac{3}{2}\sin\left(\frac{4}{9}\right)\left[\frac{r^{10}}{10c^8} - \frac{r^6}{3c^4} + \frac{r^2}{2}\right] & for\ |r| \le c \\ \frac{3}{2}\sin\left(\frac{16c^2}{135}\right) & for\ |r| \le c \end{cases} \qquad (16)$$

Differentiating Eq (16) w.r.t $\hat{\beta}_j$ we get the ψ-function as

$$\psi(r) = \begin{cases} r\left(\frac{3}{2}\right)\left[1-\left(\frac{r}{c}\right)^4\right]^2 \sin\left\{\frac{2}{3}\left[1-\left(\frac{r}{c}\right)^4\right]^2\right\} & for\ |r| \le c \\ 0 & for\ |r| \le c \end{cases} \qquad (17)$$

Dividing $\psi(r_i)$ by residual, we obtain the weight function as

$$w(r) = \begin{cases} \left(\frac{3}{2}\right)\left[1-\left(\frac{r}{c}\right)^4\right]^2 \sin\left\{\frac{2}{3}\left[1-\left(\frac{r}{c}\right)^4\right]^2\right\} & for\ |r| \le c \\ 0 & for\ |r| \le c \end{cases} \qquad (18)$$

The graphs of the objective ρ-function, ψ-function, and weight function are shown in Fig 1A, 1B, and 1C, respectively.

Fig 1. Graphs of the functions of Uk's M-estimator (A) Objective function, (B) Ψ-function and (C) weight function

3.2. Proposed Estimator

Motivated from the estimators [13,15,18,19] in literature and by using the Uk's Redescending M-Estimator [1], the proposed estimator is defined as follows:

$$\bar{y}_{UK_i} = \frac{\bar{y} + b_{UK}\left(\bar{X} - \bar{x}\right)}{\left(\beta_j \bar{x} + \gamma_i\right)}\left(\beta_j \bar{X} + \gamma_i\right),\ i = 1,2,3,4,5 \tag{19}$$

The MSE equation of the estimator in the Eq (19) is obtained by

$$MSE\left(\bar{y}_{UK_i}\right) \cong \theta\left(R_j^2 S_x^2 + 2B_{UK} R_j S_x^2 + B_{UK}^2 S_x^2 - 2R_j S_{xy} - 2B_{UK} S_{xy} + S_y^2\right) \quad \text{for } i = 1,2,3,4,5 \tag{20}$$

where B_{UK} is calculated from the objective function in the Eq (16) and $R_1 = \bar{Y}/\bar{X}$

$R_2 = \bar{Y}/\left(\bar{X} + C_x\right)$, $R_3 = \bar{Y}/\left(\bar{X} + \beta_2(x)\right)$, $R_4 = \beta_2(x)\bar{Y}/\left(\beta_2(x)\bar{X} + C_x\right)$, and

$R_5 = C_x\bar{Y}/\left(C_x\bar{X} + \beta_2(x)\right)$.

To evaluate the efficiency of the proposed ratio estimator, MSE equations of the estimators will be compared in Section 3.3.

3.3. Efficiency comparisons

For the theoretical comparisons of the proposed estimator with other estimators, it is first necessary to compare it with the traditional estimator proposed by Kadilar and Cingi [13].

$$MSE\left(\bar{y}_{KC_j}\right) > MSE\left(\bar{y}_{UK_i}\right)$$

$$2BR_jS_x^2 + B^2S_x^2 - 2BS_{xy} - 2B_{UK}R_jS_x^2 - B_{UK}^2S_x^2 + 2B_{UK}S_{xy} > 0$$

$$2R_j\left(B - B_{UK}\right) + \left(B^2 - B_{UK}^2\right) - 2b\left(B - B_{UK}\right) > 0$$

where *b* is LS slope obtained by the OLS method.

$$\left(B - B_{UK}\right) + \left(2R_j + B + B_{UK} - 2b\right) > 0 \qquad (21)$$

From Eq (21), it is possible to compare the estimators to a general formula with B^* which can be B, B_{HM}, B_{RM}, and B_{NM} as follows:

(1) $B^* > B_{UK}$

(2) $B^* + B_{UK} > 2b - 2R_j$

If the given Conditions (1) and (2) are satisfied, the proposed estimator is the most efficient estimator.

4. Numerical comparisons

4.1. Real data studies

To prove the efficiency of the proposed estimators, real-life data examples are considered. For this strategy, we use two different datasets. The first real dataset is the apple production data taken from the Black Sea Region in Turkey [15]. Apple production in tons is taken as a study variable and the number of trees (1 unit= 1000 trees) in 204 villages is taken as an auxiliary variable. Table 1 shows the population parameters for the first real dataset. Fig 2 shows the scatter plot of the data where outliers can be seen clearly.

Table 1. Parameters of apple production dataset

N = 204	$\beta_2(x)$ =29.77	ρ =0.713
n = 30	C_x =1.717	B= 4.165872
$\bar{Y}$ =966.96	R_{KC1} =3.656933	B_{KC}= 3.556434
$\bar{X}$ =264.42	R_{KC2} =3.633339	B_{NM} = 2.50765
S_y =2389.77	R_{KC3} =3.286817	B_{UK} = 2.497468
S_x =454.03	R_{KC4} =3.656136	
S_{xy} =773727.8	R_{KC5} =3.431872	

Fig 2. Scatter plot of apple dataset

For the comparison, the reference estimator is $\bar{y}_{KCi}$ traditional ratio estimator. Percent relative efficiency is computed by using

$$PRE\left(\bar{y}_{pi}\right)=\frac{MSE\left(\bar{y}_{KC_j}\right)}{MSE\left(\bar{y}_{*_j}\right)}*100; \quad j=1,2,3,4,5 \qquad (22)$$

where *=*HM*, *RM*, *NM* and *UK*. 10000 samples of n=30 size were drawn from the population with N=204 and the PREs were calculated with the help of the Eq (22) and the obtained values are given in Table 2. The best predictors are marked with "*" in the table.

Table 2. PREs for the apple production dataset (%)

Reference	$\bar{y}_{HMj}$	$\bar{y}_{NMj}$	$\bar{y}_{UKj}$
$\bar{y}_{KC1}$	125.0948	144.6571	145.0107*
$\bar{y}_{KC2}$	125.1424	144.3392	144.6912*
$\bar{y}_{KC3}$	125.5125	142.014	142.3519*
$\bar{y}_{KC4}$	125.0969	144.629	144.9825*
$\bar{y}_{KC5}$	125.2602	142.9627	143.3072*

The second real dataset concerning the U.S. State Public-School Expenditures is used. This data consists of fifty-one observations indicating the per-capita income in dollars and per-capita education expenditure in dollars for the U. S. states in 1970 [20]. The per-capita income is taken as the study variable and per-capita education expenditure is taken as an auxiliary variable. The original data was free from outliers. For this reason, a 7% outlier was added as in Raza [18]. The scatter plots of the original and outlier-added data are given within the Fig 3. The parameters of each population are given in Table 3. All of the calculations have been made as in the first real dataset and the obtained PRE values are given in Table 4. The best estimators are marked with “*”. As shown in Table 4, we see that the proposed estimators are quite efficient estimators according to other estimators, especially for the outlier-added data.

Table 3. Parameters of public school expenditures real dataset

**Original**	N = 51<br>n = 30<br>$\bar{Y} = 196.3137$<br>$\bar{X} = 3225.294$<br>S_y= 46.45449<br>S_x= 560.026<br>S_{xy}= 17367.51	$\beta_2(x)$= 2.288739<br>C_x= 0.1736356<br>R_{KC1}= 0.06086692<br>R_{KC2}= 0.06086365<br>R_{KC3}= 0.06082376<br>R_{KC4}= 0.06086549<br>R_{KC5}= 0.06061918	ρ= 0.6675773<br>B= 0.05537594<br>B_{KC}= 0.05353533<br>B_{NM}= 0.05739941<br>B_{UK}= 0.05033515
**Outlier-added**	N = 55<br>n = 30<br>$\bar{Y} = 215.0182$<br>$\bar{X} = 3321.618$<br>S_y= 81.4779<br>S_x= 642.8723<br>S_{xy}= 38997.47	$\beta_2(x)$= 2.40009<br>C_x= 0.1935419<br>R_{KC1}= 0.06473296<br>R_{KC2}= 0.06472919<br>R_{KC3}= 0.06468622<br>R_{KC4}= 0.06473139<br>R_{KC5}= 0.06449219	ρ= 0.7445123<br>B= 0.09346485<br>B_{KC}= 0.07535126<br>B_{NM}= 0.05532234<br>B_{UK}= 0.05060727

Table 4. PREs for the public school expenditures real dataset (%)

Reference	Original			Outlier of 7%		
	$\bar{y}_{HMj}$	$\bar{y}_{NMj}$	$\bar{y}_{UKj}$	$\bar{y}_{HMj}$	$\bar{y}_{NMj}$	$\bar{y}_{UKj}$
$\bar{y}_{KC1}$	104.4891	97.94668	104.8218*	125.3495	146.8591	151.5663*
$\bar{y}_{KC2}$	104.4891	97.94663	104.8218*	125.349	146.8564	151.563*
$\bar{y}_{KC3}$	104.4891	97.94605	104.8212*	125.3436	146.8273	151.5265*
$\bar{y}_{KC4}$	104.4891	97.94666	104.8218*	125.3492	146.8579	151.5649*
$\bar{y}_{KC5}$	104.4892	97.94336	104.8185*	125.3182	146.697	151.3624*

Fig 3. Scatter plot of public school expenditures dataset

A comparison of the proposed estimators with each other for all real datasets used is summarized in Fig 4. Accordingly, it can be inferred that among the proposed estimators, $\bar{y}_{UK5}$ is the most effective one in general.

Fig 4. Comparison plot of the proposed estimators

In all of the various real datasets used, the proposed estimator is found to be the most efficient estimator. Theoretically, using the information given in Tables 1 and 3, it is shown that the condition of Eq (21) is satisfied.

4.2. Simulation study

The simulation study is also conducted to check the superiority of the proposed estimator. For this purpose, data is generated from the normal distribution by using the R software. Results are calculated from the 10000 SRS (without replacement) samples. Efficiency is compared for 20, 30, 40, and 50 sample sizes of *n*. Also, we consider the outlier rates as 0.05 and 0.1. The following regression model is used to generate data for the simulation study:

$$y_i = \alpha + bx_i + e_i$$

where e_i refers to residuals and α=2, b=1.

To verify the efficiency of the proposed estimator, 95% of the study variable is generated using N(20,10), and 5% of the variable is generated using N(50,10) for outlier data. Residuals are generated using with the same ratio of N(0,1) and N(30,1), respectively. The tuning constants were taken as 1.5 for Huber, and 3 for NM and UK as suggested. Note that this simulation study is repeated for 10% outlier data as well. The calculated B coefficients are given in Table 5. PRE values were calculated using Eq (22) and the results are given in Table 6. The best predictors are marked as before. Note that the PRE values of the proposed estimators are also presented in Fig 5.

Table 5. *B* coefficients for M-Estimators under different rate of outliers and sample sizes

n	Outlier Rate: 5%				Outlier Rate: 10%			
	B	B_{HM}	B_{NM}	B_{UK}	B	B_{HM}	B_{NM}	B_{UK}
**20**	1.319016	1.026611	0.990509	0.982837	1.473978	1.136479	1.014438	1.002837
**30**	1.239935	1.006161	0.996079	0.992051	1.463691	1.109682	1.011521	1.002521
**40**	1.308943	0.992054	0.983044	0.979714	1.459319	1.096287	1.009964	1.002419
**50**	1.266481	1.012449	1.003065	1.002283	1.456715	1.089494	1.008753	1.002176

Table 6. PREs of robust estimators by simulation (%)

n	Reference	Outlier Rate: 5%			Outlier Rate: 10%		
		$\bar{y}_{HMj}$	$\bar{y}_{NMj}$	$\bar{y}_{UKj}$	$\bar{y}_{HMj}$	$\bar{y}_{NMj}$	$\bar{y}_{UKj}$
**20**	$\bar{y}_{KC1}$	164.6673	169.9909	173.6181*	173.1882	218.059	222.5642*
	$\bar{y}_{KC2}$	166.6329	172.1144	175.8659*	175.3731	221.7894	226.4527*
	$\bar{y}_{KC3}$	175.278	180.8811	185.0921*	186.0025	236.7097	241.8854*
	$\bar{y}_{KC4}$	165.271	170.6599	174.3265*	173.7791	219.1975	223.7529*
	$\bar{y}_{KC5}$	180.9139	186.540	190.969*	192.1066	244.8612	250.1742*
**30**	$\bar{y}_{KC1}$	147.2726	149.2286	151.9068*	181.889	218.3278	221.8743*
	$\bar{y}_{KC2}$	148.6053	150.6146	153.3773*	184.3227	221.9861	225.6531*
	$\bar{y}_{KC3}$	154.7781	156.8499	159.9852*	196.397	238.1077	242.2264*
	$\bar{y}_{KC4}$	147.6858	149.664	152.3682*	182.5465	219.3851	222.9674*
	$\bar{y}_{KC5}$	159.5052	161.6217	165.0045*	203.9167	247.6544	251.9397*
**40**	$\bar{y}_{KC1}$	163.6049	169.3674	171.8475*	186.5542	218.4778	221.4503*
	$\bar{y}_{KC2}$	165.4164	171.3515	173.9109*	189.1126	222.084	225.1561*
	$\bar{y}_{KC3}$	174.9103	181.4948	184.427*	201.9794	238.766	242.2344*

	$\bar{y}_{KC4}$	164.1029	169.9191	172.4215*	187.2422	219.4918	222.493*
	$\bar{y}_{KC5}$	181.7143	188.6655	191.8099*	210.3214	249.0572	252.6816*
**50**	$\bar{y}_{KC1}$	153.6796	157.5499	159.5689*	188.9728	218.8553	221.4482*
	$\bar{y}_{KC2}$	155.1604	159.1438	161.2252*	191.5885	222.4366	225.1154*
	$\bar{y}_{KC3}$	163.0814	167.5228	169.9198*	204.9737	239.6492	242.6888*
	$\bar{y}_{KC4}$	154.0893	157.9946	160.031*	189.6669	219.8365	222.4534*
	$\bar{y}_{KC5}$	169.1521	173.8924	176.4997*	213.8642	250.5479	253.7377*

Fig 5. PREs of the proposed estimator against $\bar{y}_{UK1}$

5. Conclusion

In the simple random sampling, under the determined conditions, the ratio estimators are employed to estimate the population mean efficiently. On the other hand, M-estimators are developed in the case that the data contains outliers. It has been seen from the studies in the literature that more effective results are obtained as a result of combining the ratio estimators and the M-estimators in the presence of outliers. Our results require additional precision; however, the outliers violate the OLS assumptions and do not produce good results. We present a Uk's redescending M-estimator-based ratio estimator to solve this problem. To support the proposed estimators, real-life data examples and a simulation study are conducted and they prove the efficiency of the proposed estimator.

In real data studies, it is noteworthy that the proposed estimators are more effective than the others. It was observed that the efficiency of robust estimators increased as the number of outliers increased in the data. The most striking point observed in real data studies is on the original public school expenditures real dataset. The efficiency of the $\bar{y}_{NMj}$ estimators on this real dataset without outliers is even lower than the reference that is a non-robust ratio estimator. In contrast, the proposed estimator is still the most efficient estimator.

The simulation results are also obtained in a way that supports the real data study. As the number of outliers increases, the efficiency of robust estimators increases and the most effective one is the proposed estimators again. In both real data and simulation studies, it is an advantage in terms of the usability of the proposed estimator that the necessary conditions are provided for the estimator to be effective. Therefore, the most efficient estimator in all numerical studies is the proposed estimator. When the estimators were compared among themselves, it was seen that $\bar{y}_{UK5}$ was superior to the others. However, this estimator includes more population parameters of the auxiliary variable. However, if only the mean of the auxiliary variable is known, the $\bar{y}_{UK1}$ estimator can be used as a more effective alternative than other estimators in the literature.

For future study, examining the proposed estimator, under other sampling methods, such as systematic, stratified, or ranked set sampling, can be considered as in the SRS method. Alternatively, different ratio estimators based on Uk's redescending M-estimator can also be suggested.

Disclosure statement

No potential conflict of interest was reported by the author(s).

References

[1] Khalil U, Ali A, Khan DM, Khan SA, Qadir F. Efficient Uk's Redescending M-Estimator for Robust Regression. Pakistan Journal of Statistics. 2016;32(2): 125–138.

[2] Beaton AE, Tukey JW. The Fitting of Power Series, Meaning Polynomials, Illustrated on Band-Spectroscopic Data. Technometrics. 1974;16(2): 147–185.

[3] Andrews DF. A Robust Method forMultiple Linear Regression. Technometrics. 1974;16(4): 523–531.

[4] Andrews DF, Hampel FR. Robust Estimates of Location: Festschrift Wilks (Samuel S): Survey and Advances. Princeton University Press; 2015.

[5] Qadir MF. Robust Method for Detection of Single and Multiple Outliers. Sci. Khyber. 1996;9(2): 135–144.

[6] Ali A, Qadir MF. A Modified M-Estimator for the Detection of Outliers.Pakistan J. Stat. Oper. Res. 2005; 1(1): 49–64.

[7] Ali A, Khan SA, Khan DM, Khalil U. A New Efficient Redescending M-Estimator : Alamgir Redescending M- Estimator. Res. J. Recent Sci. 2013; 2(8): 79–91.

[8] Hampel FR, Ronchetti EM, Rousseeuw P, Stahel WA. Robust Statistics: the Approach based on Influence Functions. Wiley-Interscience, New York; 1986.

[9] Huber PJ. Robust Estimation of a Location Parameter. Ann. Math. Stat. 1964;35(1): 73–101.

[10] Huber PJ, Ronchetti EM. Robust Statistics. John Wiley & Sons, New York; 1981.

[11] Ullah I, Qadir MF, Ali A. Insha's Redescending M-estimator for Robust Regression: A Comparative Study. Pakistan J. Stat. Oper. Res. 2006; 2(2): 135-144.

[12] Noor-ul-amin M, Asghar SUD, Sanaullah A, Shehzad MA. Redescending M-Estimator for Robust Regression. Journal of Reliability and Statistical Studies. 2018;11(2): 69–80.

[13] Kadilar C, Cingi H. Ratio Estimators inSimple Random Sampling. Appl. Math. Comput. 2004;151(3): 893–902.

[14] Ray SK, Singh RK. Difference-cum-ratio type estimators. J. Ind. Stat. Assoc. 1981; 19: 147–151.

[15] Kadilar C, Candan M, Cingi H. Ratio Estimators Using Robust Regression. Hacettepe Journal of Mathematics and Statistics. 2007;36(2): 181–188.

[16] Sisodia BVS, Dwivedi VK. Modified Ratio Estimator Using Coefficient of Variation of Auxiliary Variable. Journal-Indian Society of Agricultural Statistics.. 1981;33: 13-18.

[17] Upadhayaya LN, Singh HP. Use of Transformed Auxiliary Variable in Estimating the Finite Population Mean. Biometrical Journal. 1999;41(5): 627–636.

[18] Raza A, Noor-ul-amin M, Hanif M. Regression-in-Ratio Estimators in The Redescending M-Estimator. Journal of Reliability and Statistical Studies. 2019;12(2): 1–10.

[19] Noor-ul-amin M, Asghar S, Sanaullan A. Ratio Estimators in the Presence of Outliers Using Redescending M-Estimator. Proceedings of the National Academy of Sciences, India Section A: Physical Sciences. 2020; 1–6.

[20] Fox J, Applied Regression Analysis and Generalized Linear Models. Third Edition. Sage, Los Angeles; 2016.

Fig 1 Click here to access/download;Figure;Fig 1.eps

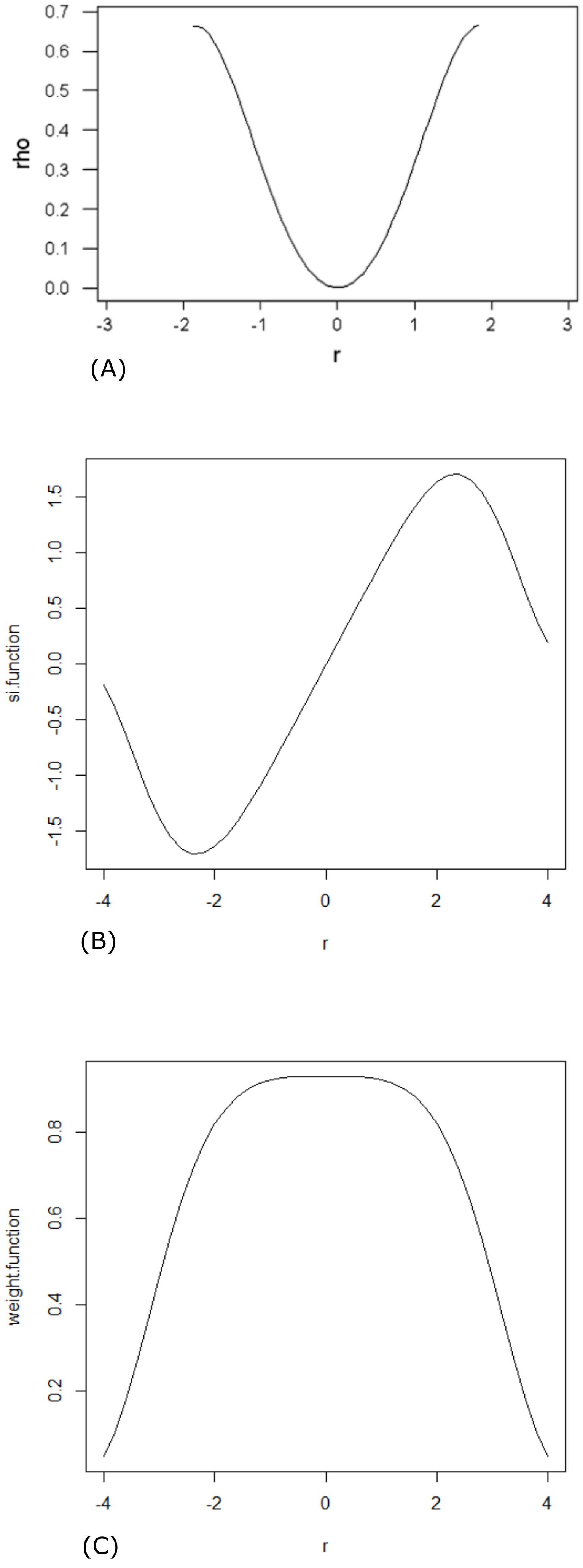

Fig 2 Click here to access/download;Figure;Fig 2.eps

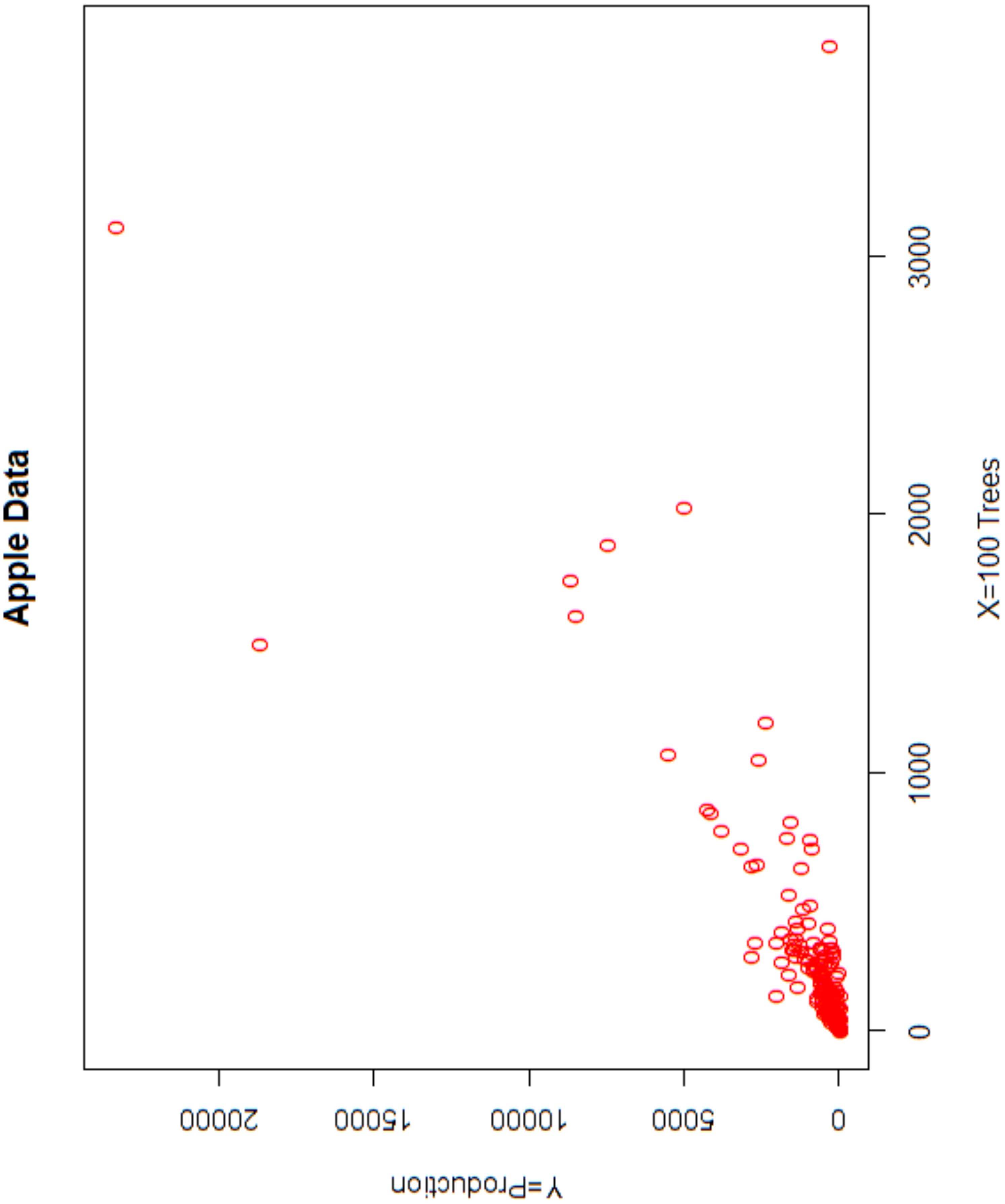

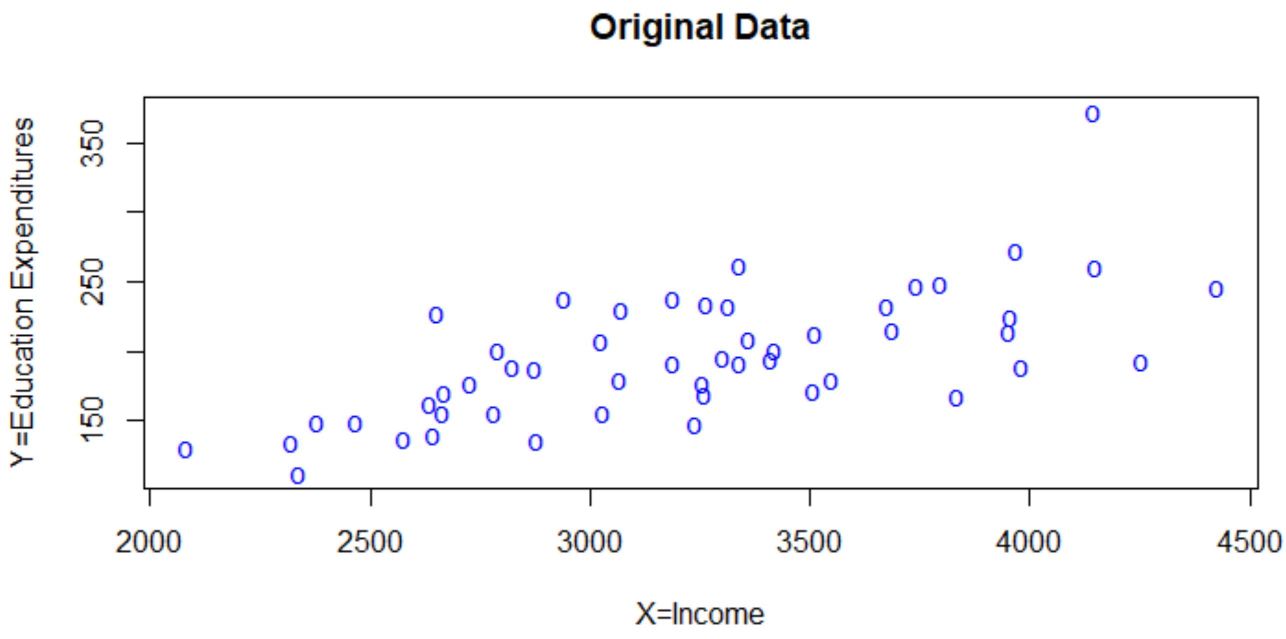
Original Data
Y=Education Expenditures
150
250
350
2000
2500
3000
3500
4000
4500
X=Income

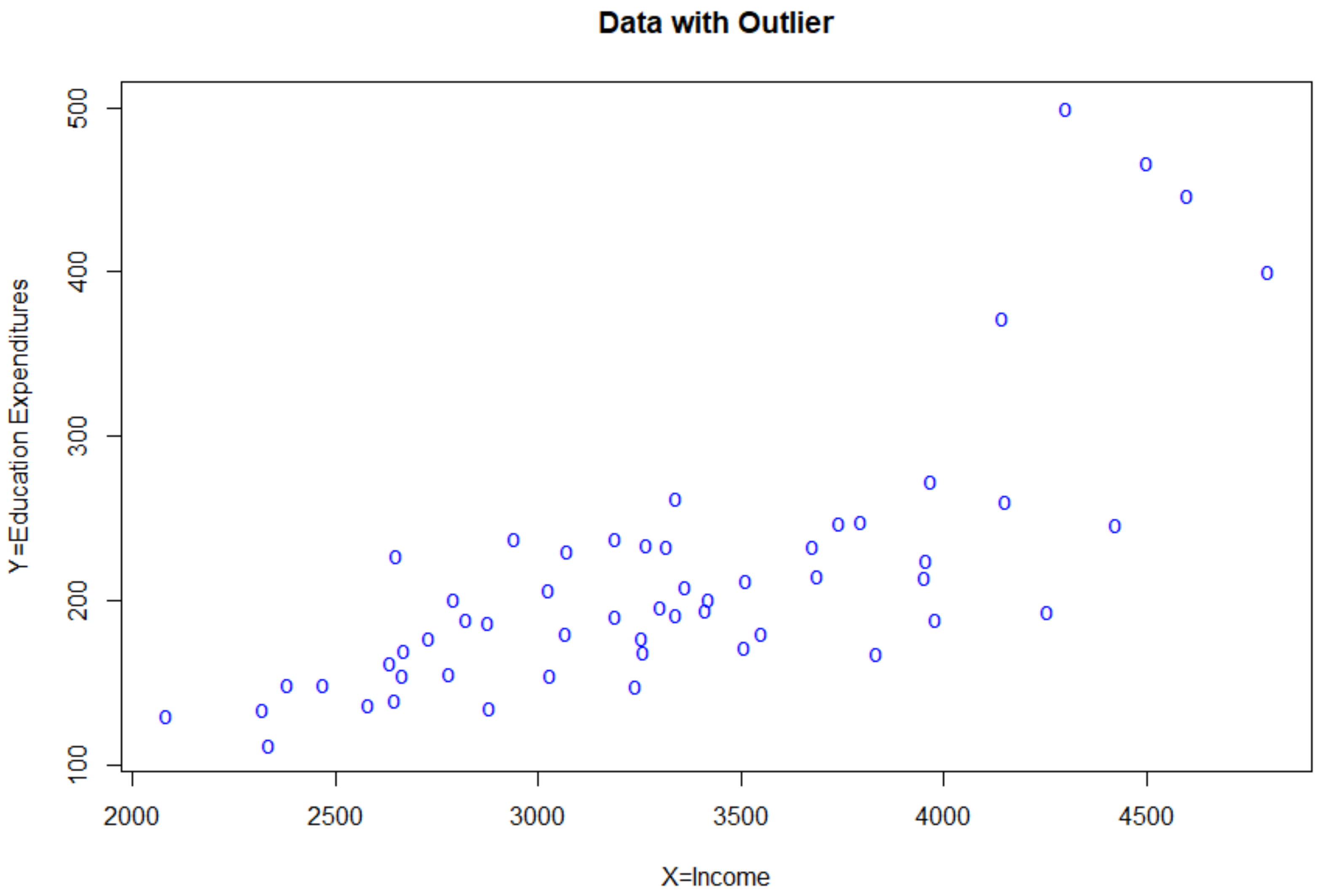
Data with Outlier
Y=Education Expenditures
100
200
300
400
500
2000
2500
3000
3500
4000
4500
X=Income

Fig 4 Click here to access/download;Figure;Fig 4.eps

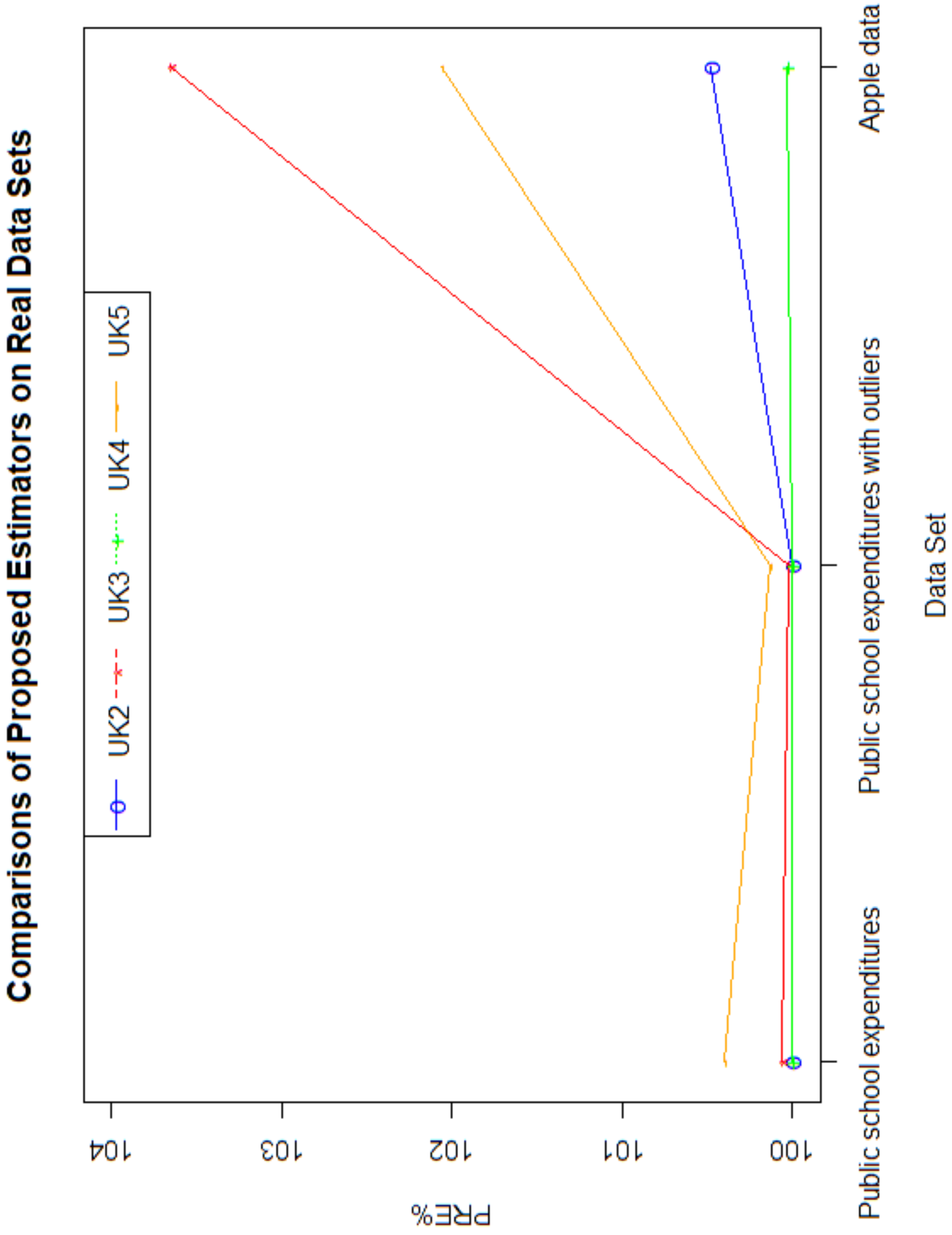

Fig 5

Click here to access/download;Figure;Fig 5.eps

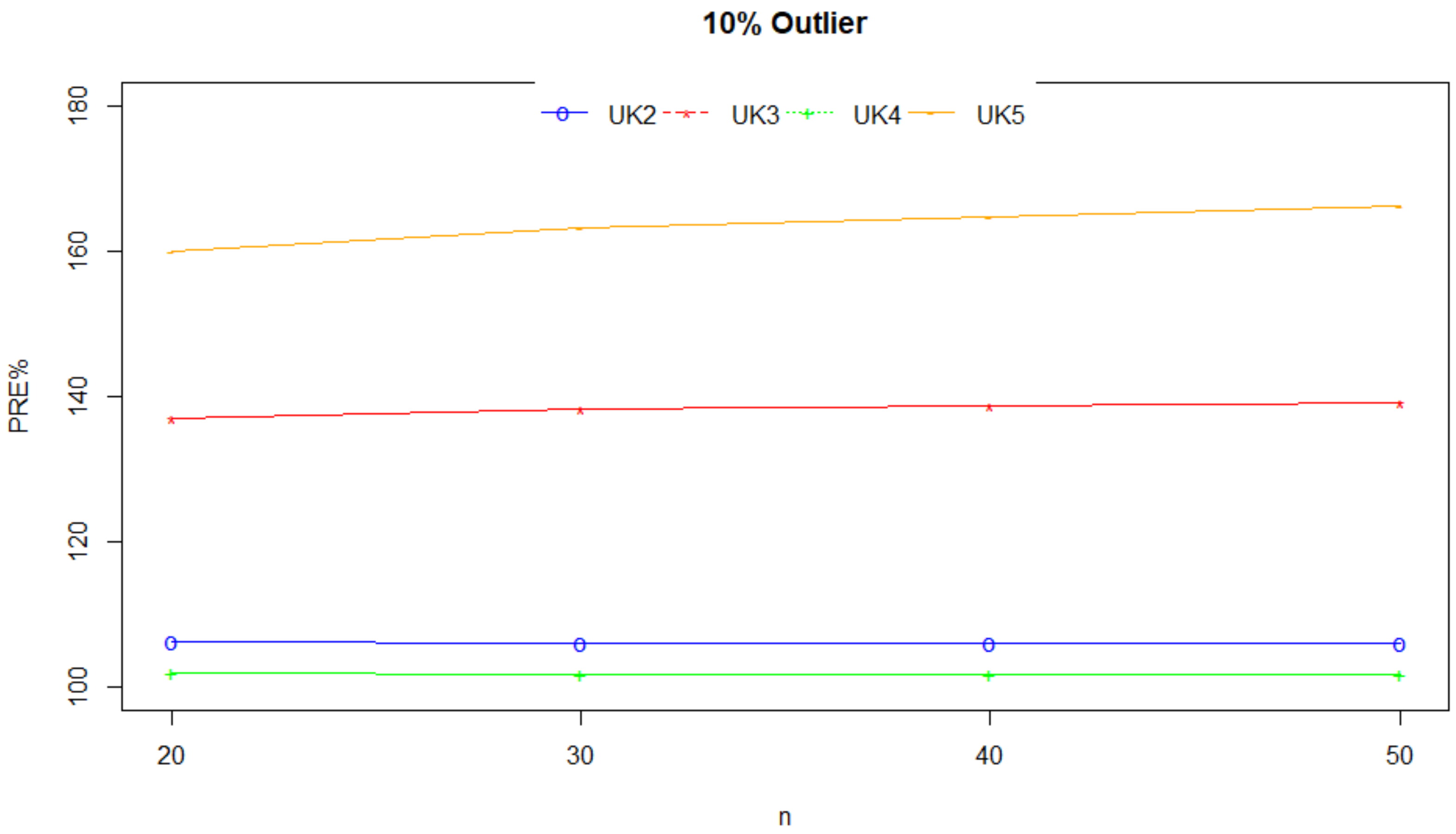

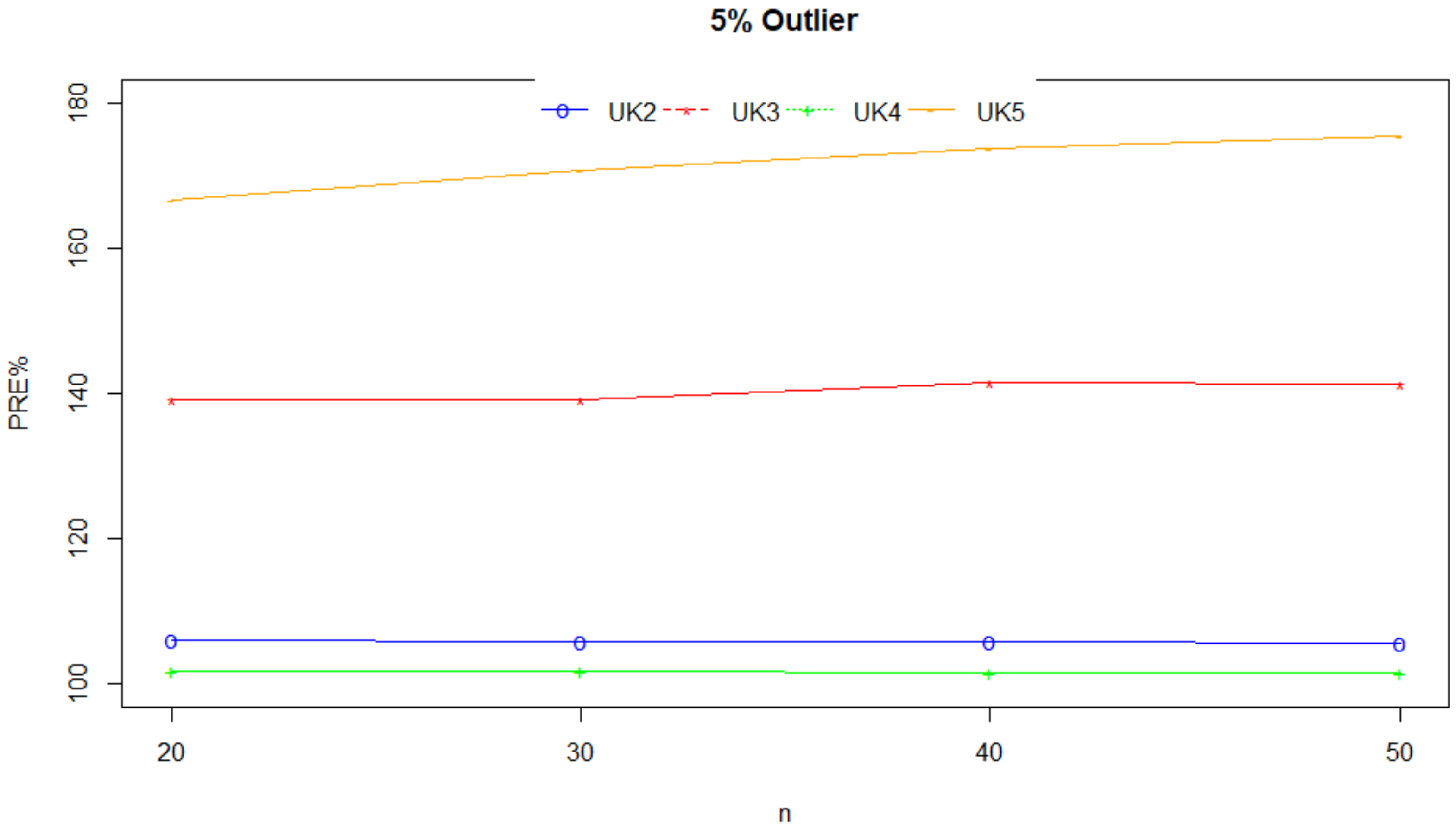

Supplement: Attachment — Submitted filename: PONE-D-22-27634_comments.pdf [file pone.0278868.s003.pdf]
