# Supplementary material for: Improved regression in ratio type estimators based on robust M-estimation
Source: PLoS One. 2022 Dec 12;17(12):e0278868. doi: 10.1371/journal.pone.0278868 (PMC9744333; doi:10.1371/journal.pone.0278868)
Supplement: Attachment — Submitted filename: Response to Reviewers.pdf [file pone.0278868.s004.pdf]

RESPONSE TO REVIEWERS

<u>Reviewer #1:</u> To improve the novelty of the manuscript, some other objective functions should also be used.

The real data application to demonstrate the application of the proposal should be the part of the paper. The overall language of the paper needs an improvement.

Dear Reviewer I ,

Thank you for your review. We started this work with the aim of developing the estimator proposed by Noor-ul Amin et al.(2020). The methods proposed before 2020 have already been compared in that article and it has been shown that the proposed ratio estimator is better than the estimators in the literature. We consider it sufficient to show that our proposed estimator is better than the proposed method in 2020. However, we expanded the Introduction section and gave the existing estimators.

In our study, there are already real data studies in addition to the simulation study. If what you are talking about is solving numerical calculations step by step, this is quite difficult and complex due to objective functions. For this reason, we did all the operations on the R program and shared the codes with the journal.

We have worked on the changes you mentioned. We hope that the revised version of the work will satisfy you.

Regards.

<u>Reviewer #2</u>: After reviewing the related literature in detail, authors develop a new family of the robust ratio estimators using the Uk’s redescending M-estimator successfully. In both application and simulation study, it is found that the proposed family is the most efficient estimator according to estimators in literature. As authors mention, these results support the theoretical efficiency condition obtained in (21). Authors derives two conditions from (21) but these conditions should be corrected as follows:

From (21),

B-B_UK+2R_j+B+B_UK-2b>0,

$2B+2R_j-2b>0$,

$R_j>b-B$.

Authors should evaluate the results of application and simulation based on the theoretical efficiency condition: $R_j>b-B$.

After revising the manuscript according to the mentioned correction, the manuscript can be published in Plos One.

Dear Reviewer II ,

Thanks for your valuable review. The expression "+" is misspelled in the formula in the equation you specified. The same equation has been written more clearly and corrected.

Regards.

<u>Reviewer #3:</u> The authors propose a new robust ratio type estimator using the Uk’s redescending M-estimator is proposed for the estimation of the finite population mean in the simple

24 random sampling (SRS) when there are outliers in the dataset. The properties of the proposed estimators are obtained and compared with the properties of the existing estimators. The theoretical results are enhanced empirically.

Although, theoretical and computational results are found satisfactory but, the paper is not suitable for publication in its current form. It needs revision under the comments highlighted in the pdf file attached in the attachment section.

Dear Reviewer III ,

Thanks for your careful review and advice. The corrections you requested have been made. The revised version of the paper has been submitted as a "Manuscript" file, and you can see changes with the highlighted version on the "Revised Manuscript with Track Changes" file your notes have been answered on “PONE-D-22-27634 response to comments” pdf file.

Regards.
