# Supplementary material for: Improved regression in ratio type estimators based on robust M-estimation
Source: PLoS One. 2022 Dec 12;17(12):e0278868. doi: 10.1371/journal.pone.0278868 (PMC9744333; doi:10.1371/journal.pone.0278868)
Supplement: Attachment — Submitted filename: reportplosone.docx [file pone.0278868.s002.docx]

**Referee report:**

After reviewing the related literature in detail, authors develop a new family of the robust ratio estimators using the Uk’s redescending M-estimator successfully. In both application and simulation study, it is found that the proposed family is the most efficient estimator according to estimators in literature. As authors mention, these results support the theoretical efficiency condition obtained in (21). Authors derives two conditions from (21) but these conditions should be corrected as follows:

From (21),

$$B-B_{UK}+2R_{j}+B+B_{UK}-2b>0,$$

$$2B+2R_{j}-2b>0,$$

$$R_{j}>b-B.$$

Authors should evaluate the results of application and simulation based on the theoretical efficiency condition: $R_{j}>b-B.$

After revising the manuscript according to the mentioned correction, the manuscript can be published in Plos One.
