# Supplementary material for: Improved regression in ratio type estimators based on robust M-estimation
Source: PLoS One. 2022 Dec 12;17(12):e0278868. doi: 10.1371/journal.pone.0278868 (PMC9744333; doi:10.1371/journal.pone.0278868)
Supplement: Attachment — Submitted filename: Review report.docx [file pone.0278868.s005.docx]

**Review report**

**Title: Improved regression in ratio type estimators based on robust** **M-estimation**

The authors have successfully incorporated the comments; however, the introduction section needs to be revised by incorporating the latest related references published till date. The following references can also be added to enhance the readability of the paper.

Bhushan, S. and Kumar, A. (2022). Novel log type class of estimators under ranked set sampling. Sankhya B, 84, 421-447. https://doi.org/10.1007/s13571-021-00265-y

Bhushan, S., Kumar, A., Shahab, S., Lone, S.A. and Almutlak, S.A. (2022). Modified class of estimators using ranked set sampling. Mathematics, 10, 3921, 1-13

Bhushan, S., Kumar, A. and Lone, S.A. (2022). On some novel classes of estimators under ranked set sampling. AEJ-Alexandria Engineering Journal, 61, 5465-5474. https://doi.org/10.1016/j.aej.2021.11.001.

Bhushan, S., Kumar, A., Pandey, A.P. and Singh, S. (2022). Estimation of population mean in presence of missing data under simple random sampling. Communications in Statistics - Simulation and computation. https://doi.org/10.1080/03610918.2021.2006713

Bhushan, S., Kumar, A. and Singh, S. (2021). Some efficient classes of estimators under stratified sampling. Communications in Statistics - Theory and Methods, 1-30. DOI:10.1080/03610926.2021.1939052.

Bhushan, S., Kumar, A., Akhtar, M.T. and Lone. S.A. (2022). Logarithmic type predictive estimators under simple random sampling. AIMS Mathematics, 7(7), 11992-12010.

Bhushan, S., Kumar, A., Shahab, S., Lone. S.A. and Akhtar, M.T. (2022). On efficient estimation of population mean under stratified ranked set sampling. Journal of Mathematics, 2022(3), 1-20.

Bhushan, S., Kumar, A., Onyango, R. and Singh, S. (2022). Some improved classes of estimators in stratified sampling using bivariate auxiliary information. Journal of Probability and Statistics, 2022(2), 1-23.

Bhushan, S., Kumar, A., Singh, S. and Kumar, S. (2021). An improved class of estimators of population mean under simple random sampling. Philippine Statistician, 70(1), 33-47.
