# Supplementary material for: Improved regression in ratio type estimators based on robust M-estimation
Source: PLoS One. 2022 Dec 12;17(12):e0278868. doi: 10.1371/journal.pone.0278868 (PMC9744333; doi:10.1371/journal.pone.0278868)
Supplement: Attachment — Submitted filename: Response to Reviewers.docx [file pone.0278868.s006.docx]

**RESPONSE TO REVIEWERS**

**Reviewer #2:** Manuscript is well now. All the changes are correct as per suggestion, can be published in your reputed journal PLOS.

*Dear Reviewer II,*

*Thank you for your careful review and valuable contribution to our paper in this process.*

*Regards.*

**Reviewer #3:** The authors have successfully incorporated the comments; however, the introduction section needs to be revised by incorporating the latest related references published till date. The following references can also be added to enhance the readability of the paper.

Bhushan, S. and Kumar, A. (2022). Novel log type class of estimators under ranked set sampling. Sankhya B, 84, 421-447. https://doi.org/10.1007/s13571-021-00265-y

Bhushan, S., Kumar, A., Shahab, S., Lone, S.A. and Almutlak, S.A. (2022). Modified class of estimators using ranked set sampling. Mathematics, 10, 3921, 1-13

Bhushan, S., Kumar, A. and Lone, S.A. (2022). On some novel classes of estimators under ranked set sampling. AEJ-Alexandria Engineering Journal, 61, 5465-5474. https://doi.org/10.1016/j.aej.2021.11.001.

Bhushan, S., Kumar, A., Pandey, A.P. and Singh, S. (2022). Estimation of population mean in presence of missing data under simple random sampling. Communications in Statistics - Simulation and computation. https://doi.org/10.1080/03610918.2021.2006713

Bhushan, S., Kumar, A. and Singh, S. (2021). Some efficient classes of estimators under stratified sampling. Communications in Statistics - Theory and Methods, 1-30. DOI:10.1080/03610926.2021.1939052.

Bhushan, S., Kumar, A., Akhtar, M.T. and Lone. S.A. (2022). Logarithmic type predictive estimators under simple random sampling. AIMS Mathematics, 7(7), 11992-12010.

Bhushan, S., Kumar, A., Shahab, S., Lone. S.A. and Akhtar, M.T. (2022). On efficient estimation of population mean under stratified ranked set sampling. Journal of Mathematics, 2022(3), 1-20.

Bhushan, S., Kumar, A., Onyango, R. and Singh, S. (2022). Some improved classes of estimators in stratified sampling using bivariate auxiliary information. Journal of Probability and Statistics, 2022(2), 1-23.

Bhushan, S., Kumar, A., Singh, S. and Kumar, S. (2021). An improved class of estimators of population mean under simple random sampling. Philippine Statistician, 70(1), 33-47.

*Dear Reviewer III,*

*Thank you for your careful review and valuable contribution to our paper in this process. The latest related references you mentioned in your comments have been added to the "Introduction" section of the manuscript.*

*Regards.*

**RESPONSE TO ACADEMIC EDITOR**

**ACADEMIC EDITOR:** Revise your manuscript according to reviewers comments. Also, revise your introduction section by mentioning the latest related publish papers. The following papers can also be discussed.

Shahzad U, Alnoor NH, Hanif M, Sajjad I, Anas MM. Imputation based mean estimators in case of missing data utilizing robust regression and variance-covariance matrices. Communications in Statistics Simulation and Computation. 2020a. 32.

Shahzad U, Alnoor NH, Hanif M,Sajjad I,Anas MM. Quantileregression-ratio-type estimators for mean estimation under complete and partial auxiliary information. Scientia Iranica. 2020b. https://doi.org/10.24200/sci.2020.54423.3744/

*Dear Academic Editor,*

*Thank you for your contribution to our paper. We revised the manuscript according to the reviewers' comments. Also, the cited references you pointed out have been added to the "Introduction" section.*

*Regards.*
